# Texture analysis of intermediate-advanced hepatocellular carcinoma: prognosis and patients' selection of transcatheter arterial chemoembolization and sorafenib

**DOI:** 10.18632/oncotarget.13675

**Published:** 2016-11-29

**Authors:** Sirui Fu, Shuting Chen, Changhong Liang, Zaiyi Liu, Yanjie Zhu, Yong Li, Ligong Lu

**Affiliations:** ^1^ Department of Interventional Oncology, Guangdong Provincial Cardiovascular Institute, Guangdong General Hospital, Guangdong Academy of Medical Sciences, Guangzhou, China; ^2^ Southern Medical University, Guangzhou, China; ^3^ Department of Radiology, Guangdong General Hospital, Guangdong Academy of Medical Sciences, Guangzhou, China; ^4^ Shenzhen Institutes of Advanced Technology, Shenzhen, China

**Keywords:** hepatocellular carcinoma, texture analysis, sorafenib, transcatheter arterial chemoembolization

## Abstract

Transcatheter arterial chemoembolization (TACE) and sorafenib combination treatment for unselected hepatocellular carcinoma (HCC) is controversial. We explored the potential of texture analysis for appropriate patient selection. There were 261 HCCs included (TACE group: *n* = 197; TACE plus sorafenib (TACE+Sorafenib) group *n* = 64). We applied a Gabor filter and wavelet transform with 3 band-width responses (filter 0, 1.0, and 1.5) to portal-phase computed tomography (CT) images of the TACE group. Twenty-one textural parameters per filter were extracted from the region of interests delineated around tumor outline. After testing survival correlations, the TACE group was subdivided according to parameter thresholds in receiver operating characteristic curves and compared to TACE+Sorafenib group survival. The Gabor-1-90 (filter 0) was most significantly correlated with TTP. The TACE group was accordingly divided into the TACE-1 (Gabor-1-90 ≤ 3.6190) and TACE-2 (Gabor-1-90 > 3.6190) subgroups; TTP was similar in the TACE-1 subgroup and TACE+Sorafenib group, but shorter in the TACE-2 subgroup. Only wavelet-3-D (filter 1.0) correlated with overall survival (OS), and was used for subgrouping. The TACE-5 (wavelet-3-D ≤ 12.2620) subgroup and the TACE+Sorafenib group showed similar OS, while the TACE-6 (wavelet-3-D > 12.2620) subgroup had shorter OS. Gabor-1-90 and wavelet-3-D were consistent. In dependent of tumor number or size, CT textural parameters are correlated with TTP and OS. Patients with lower Gabor-1-90 (filter 0) and wavelet-3-D (filter 1.0) should be treated with TACE and sorafenib. Texture analysis holds promise for appropriate selection of HCCs for this combination therapy.

## INTRODUCTION

Hepatocellular carcinoma (HCC) has been proven to be a leading cause of cancer-related death worldwide [1–3]. More than half of HCCs have been diagnosed in China, and according to the most well-recognized Barcelona Clinic Liver Cancer (BCLC) stage, more than 75% patients are in stage B or C [4, 5]. According to the guidelines adopted by the European Association for the Study of the Liver/European Organization for Research and Treatment of Cancer (EASL–EORTC), American Association for the Study of Liver Diseases (AASLD), and National Comprehensive Cancer Network (NCCN), transcatheter arterial chemoembolization (TACE) is a fundamental therapy for these HCCs [4–6], while the combination of TACE and sorafenib has been considered to be promising [7–10], as sorafenib may control the elevation of vascular endothelial growth factor (VEGF) caused by TACE [11].

Although the combination of sorafenib and TACE was anticipated to be a breakthrough, and some studies had proven the safety and potential efficacy of the combination [7, 12], 2 recent randomized control trails drew a contrary conclusion about this combination therapy [13, 14]. These controversies indicated that not all patients obtain a survival benefit from the combination. In addition, in some cases, TACE might be disturbed because of adverse effects (AEs) caused by sorafenib. Thus, a method allowing accurate patient selection for application of this combination is necessary. Multiples studies have sought potential factors, including clinical factors [15], biomarkers [16], and radiological characteristics [17], that could predict the efficacy of TACE or sorafenib. Although these studies provided meaningful insight in HCC prognosis, they fell short of accurate patients’ identification, particularly for the combination of TACE and sorafenib.

In recent years, radiomics has become another significant field, in addition to genomics and proteomics, in oncology [18]. As a technique that categorizes regions of interest in an image by spatial variations in pixel intensities [19], texture analysis has been widely applied in a number of different cancers [20–26]. In studies of liver diseases, texture analysis has been used for prediction of postoperative hepatic insufficiency [27] and fibrosis assessment [28]. Furthermore, in a previous study, we have proven that texture analysis was promising for HCC patient stratification for determining the suitability of liver resection vs. TACE [29]. Therefore, we considered that it might be a potential method for selecting patients for combination therapy.

To prove our hypothesis, we conducted this study in 2 steps: firstly, we tested whether texture analysis could be prognostic in HCC patients treated with TACE; secondly, we verified whether the identified textural parameters could be used in selecting patients that may be suitable for combination therapy.

## RESULTS

### Patients

Two-hundred-and-sixty-one patients were included, of which 197 were treated with TACE and 64 were treated with TACE plus sorafenib (TACE+Sorafenib). By the end-date, 150 patients in the TACE group and 58 of 64 (91%) patients in the TACE+Sorafenib had died. Fifteen of 197 (8%) patients in the TACE group were lost to follow-up of overall survival. Furthermore, 191 of 197 (97%) patients in the TACE group and 61 of 64 (95%) in the TACE+Sorafenib group had recorded PD. During treatment, there were 74 (37.6%) in the TACE group vs. 17 (26.6%) patients in the TACE+Sorafenib group received ablation (*P* = 0.149). Their demographic were shown in Table.1.

**Table 1:** Demographic and baseline characteristics of the patients

	All (*N* = 261)	TACE (*N* = 197)	Sorafenib (*N* = 64)	*P*
Age	56 (20–83)*	58(20–84)*	54 (20–79)*	0.103
Sex (N)				0.094
Male	241	185	56	
Female	20	12	8	
**BMI (kg/m^2^)**	24(15–32)*	24 (15–32)*	23 (16–33)*	0.840
**Cause of disease (N)**				1.000
HBV	195	147	48	
HCV	4	3	1	
Negative	62	47	15	
**Child–Pugh class (N)**				0.231
A	180	132	48	
B	81	65	16	
**BCLC (N)**				
AB		62	13	0.081
B		75	26	
C		60	25	
**Vascular invasion**				0.114
No	176	138	38	
Yes	85	59	26	
**Cirrhosis**				0.984
Yes	196	148	48	
No	65	49	16	
**MD (mm)**	75 (42–187)*	74 (42–187)*	77 (48–175)*	0.821
**Lesion number (N)**				0.446
N = 1	113	93	20	
N = 2	67	33	34	
N = 3	13	3	10	
N ≥ 4	68	68	0	
**Albumin (g/L)**	35 (21–48)*	35 (22–44)*	34 (21–48)*	0.568
**TBIL (μmol/L)**	20 (5–52)*	20 (8–52)*	22 (5–37)*	0.812
**Prothrombin time**	14 (12–16)*	14 (12–15)*	14 (12–16)*	0.418
**ALT (μmol/L)**	38 (10–566)*	38 (15–566)*	39 (10–236)*	0.480
**AFP (N)**				0.125
< 25μg/mL	69	57	12	
25–400 μg/mL	93	71	22	
> 400 μg/mL	99	69	30	

Abbreviations:* median (range) for data without normal distribution.

BCLC: Barcelona Clinic Liver Cancer; BMI: body mass index; HBV: hepatitis B virus; HCV: hepatitis C virus; MD: maximum diameter; TBIL: total bilirubin; ALT: alanine aminotransferase, AFP: alpha fetoprotein.

### Screening of candidate textural parameters by Cox regression analysis

For TTP, univariate Cox regression showed that 17 parameters in the Gabor filter (2 in filter 0, 5 in filter 1.0, and 10 in filter 1.5), and 16 wavelet transform (1 in filter 0, 6 in filter 1.0, and 9 in filter 1.5) had *P* values < 0.10 (Supplementary Table E1). Among candidate clinical factors, only VI/EM had a *P* value < 0.10. Six separated multivariate Cox regression analyses, including both clinical factors and textural parameters, showed that Gabor-1-90 (filter 0), Gabor-1-135 (filter 1.0), Gabor-1-135 (filter 1.5), wavelet-2-D (filter 1.0) and wavelet-3-D (filter 1.5) were significantly related to TTP. In addition, VI/EM was also related to TTP in the Cox regression analysis for wavelet transform in filter 1.0 (Supplementary Tables S2, S3).

For OS, during follow-up, 16 patients were lost to follow-up after PD; hence, OS analysis was performed using the remaining 245 patients. In this analysis, univariate Cox regression analysis showed that 17 parameters in 5 Gabor filter (all in filter 1.0) and 2 wavelet transform (all in filter 1.0) had *P* values < 0.10 (Supplementary Table S1). Among candidate clinical factors, only sex had a *P* value < 0.10. Therefore, only 2 individual multivariate Cox regression analyses that included both clinical factors and textural parameters were performed; the results showed that sex and wavelet-3-D (filter 1.0) were significantly related to OS (Supplementary Tables S2, S3).

### ROC curves

Among the selected textural parameters identified by multivariate Cox regression analyses, ROC curves were drawn to identify the thresholds. The results showed that the thresholds were 3.6190 for Gabor-1-90 (filter 0), 1.3995 for Gabor-1-135 (filter1.0), 0.5175 for Gabor-1-135 (filter 1.5), 18.3585 for wavelet-2-D (filter 1.0), 6.7515 for wavelet-2-D (filter 1.5), and 12.2620 for wavelet-3-D (filter 1.0), respectively.

### Kaplan-Meier analysis of disease progression in TACE patients

**Figure 1:**
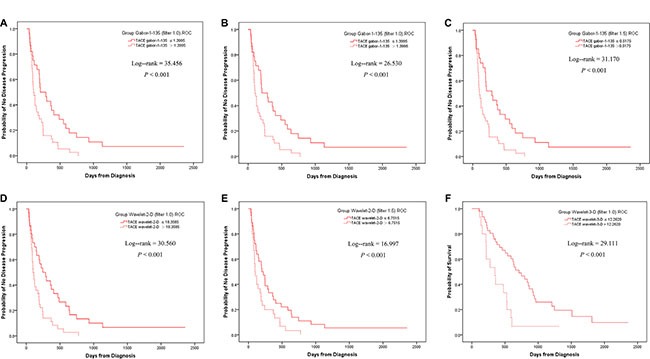
Kaplan-Meier analyses for TTP in TACE patients When separated by the ROC threshold of Gabor-1–90 at filter 0, the two subgroups had a statistical difference in TTP (**A**). Similar results could be achieved by the threshold of Gabor-1-135 at filter 1.0 (**B**), Gabor-1-135 at filter 1.5 (**C**) wavelet-2-D at filter 1.0 (**D**) and wavelet-2-D at filter 1.5 (**E**). When separated by the threshold of wavelet-3-D at filter 1.0, the two subgroups had a statistical difference in OS (**F**).

For TTP, when the TACE group was separated by the threshold of Gabor-1-90 (filter 0), the 2 subgroups were statistically significantly different (*P* < 0.001, Figure 1A). Similar results could be achieved by using Gabor-1-135 (filter 1.0), Gabor-1-135 (filter 1.5), wavelet-2-D (filter 1.0), and wavelet-2-D (filter 1.5) (Figure 1B, 1C, 1D, and 1E, Supplementary Table S4). For OS, when the TACE group was separated by the threshold of wavelet-3-D (filter 1.0), the 2 subgroups were also statistically significantly different (*P* < 0.001, Figure 1F, Supplementary Table S4).

### Kaplan-Meier analysis in all the patients

**Figure 2:**
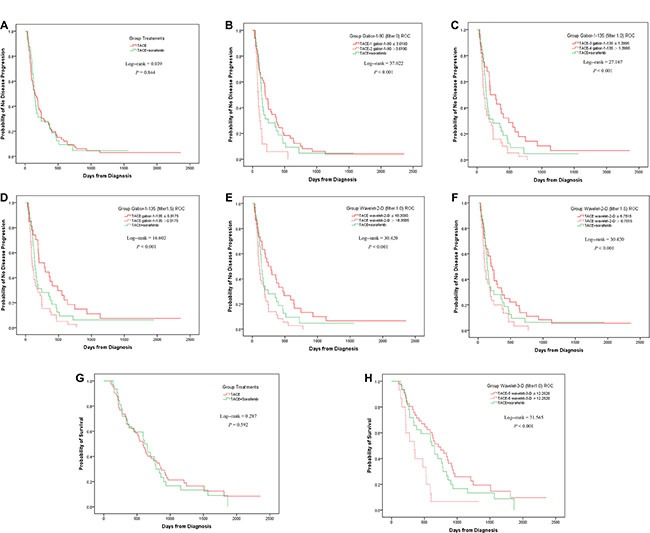
Kaplan-Meier analyses in all the patients Without subgrouping, the TACE and TACE+Sorafenib group did not had a statistical difference in TTP (**A**). When the TACE group was divided by Gabor-1-90 at filter 0 into TACE-1 and TACE-2 subgroups, the difference between the 3 groups were statistically significantly different, arising from the difference between TACE-2 and TACE+Sorafenib, but not between TACE-1 and TACE+Sorafenib (**B**). Similar results were achieved by wavelet-2-D at filter 1.5 (**F**). When TACE was separated by the threshold of Gabor-1-135 at filter 1.0 into TACE-3 and TACE-4, statistically significant differences were found in all the pairwise comparisons (**C**). Similar results were found when using Gabor-1-135 at filter 1.5 (**D**) or wavelet-2-D at filter 1.0 (**E**). Without subgrouping, the TACE and TACE+Sorafenib group did not had a statistical difference in OS (**G**). When the TACE group was separated by wavelet-3-D at filter 1.0 into TACE-5 and TACE-6, the difference between the 3 groups was statistically significant. In pairwise comparisons, there was a statistically significant difference between TACE-6 and TACE+Sorafenib, but not between TACE-5 and TACE+Sorafenib (**H**).

Without detailed grouping, the TACE and TACE+Sorafenib groups showed no statistically significant difference in either TTP or OS (Figure 2A, 2G, Supplementary Table S4). This seemed to indicate that the combination of TACE and sorafenib had limited efficacy. However, when the TACE group were separated by the identified textural parameters, different results were found, as detailed below.

For TTP, when the TACE group was divided by Gabor-1-90 (filter 0) into TACE-1 (Gabor-1-90 ≤ 3.6190) and TACE-2 (Gabor-1-90 > 3.6190) subgroups, the difference between the 3 groups (TACE-1, TACE-2, and TACE+Sorafenib) were statistically significantly different (*P* < 0.001), arising from the difference between TACE-2 and TACE+Sorafenib, but not between TACE- 1 and TACE+Sorafenib (Figure 2B). Similar results were achieved by wavelet-2-D (filter 1.5) (Figure 2F, Supplementary Table S4). When TACE was separated by the threshold of Gabor-1-135 (filter 1.0) into TACE- 3 (Gabor-1-135 ≤ 1.3995) and TACE-4 (Gabor-1-135 > 1.3995), statistically significant differences were found in all the pairwise comparisons (Figure 2C). Similar results were found when using Gabor-1-135 (filter 1.5) or wavelet-2-D (filter 1.0) (Figures 2D, 2E, Supplementary Table S4).

For OS, when the TACE group was separated by wavelet-3-D (filter 1.0) into TACE-5 (wavelet-3-D < 12.2620) and TACE-6 (wavelet-3-D > 12.2620), the difference between the 3 groups was statistically significant (*P* < 0.001). In pairwise comparisons, there was a statistically significant difference between TACE- 6 and TACE+Sorafenib, but not between TACE-5 and TACE+Sorafenib (Figure 2H, Supplementary Table S4).

### Cox regression analysis for all the patients

For TTP, based on the results of Kaplan-Meier analysis of the data from all the patients, when separated by Gabor-1-135 (filter 1.0), Gabor-1-135 (filter 1.5), or wavelet-2-D (filter 1.0), some patients with lower values may still obtain a survival benefit from the combination of TACE and sorafenib. Thus, these parameters were inferior to the Gabor-1-90 (filter 0) and wavelet-2-D (filter 1.5) in identification of the most patients that would be suitable for combination therapy. Thus, we performed the Cox regression analysis as follows.

For TTP, univariate Cox regression analysis showed that, among the candidate clinical variables and subgrouping methods, only the subgrouping methods had a *P* value < 0.10. Then, multivariate Cox regression analyses showed that subgrouping by Gabor-1-90 (filter 0) was statistically associated with TTP (*P* = 0.007), while subgrouping by wavelet-2-D (filter 1.5) identified no factor related to TTP (Table 2).

**Table 2:** Multivariate cox regression for TTP and OS in all patients

Cox model*	Factors	HR (95% CI)	*P*
**TTP**			
**Gabor filter 0 Subgroups**	TACE+sorafenib		0.002
	Gabor-1-90 ≤3.6190	0.802 (0.505–1.274)	
	Gabor-1-90 > 3.6190	2.184 (1.190–4.007)	
**Gabor filter 1. 0 Subgroups**	TACE+sorafenib		0.004
	Gabor-1-135 ≤1.3995	0.455 (0.231–0.898)	
	Gabor-1-135 > 1.3995	1.298 (0.827–2.036)	
**Gabor filter 1.5 Subgroups**	TACE+sorafenib		0.008
	Gabor-1-135 ≤0.5175	0.665 (0.390–1.137)	
	Gabor-1-135 > 0.5175	1.503 (0.930–2.431)	
**Wavelet Transform filter 1.0 Subgroups**	TACE+sorafenib		0.007
	Wavelet-2-D ≤18.3585	0.663 (0.393–1.118)	
	Wavelet-2-D > 18.3585	1.486 (0.912–2.422)	
**Wavelet Transform filter 1.5**		None identified	
**OS**			
**Wavelet Transform filter 1.0 Subgroups**	TACE+sorafenib		0.005
	Wavelet-3-D < 12.2620	0.759 (0.465–1.239)	
	Wavelet-3-D > 12.2620	2.115 (1.101–4.062)	

Abbreviations:*Seven separate multivariate cox regression analyses were performed: 6 for TTP and 1 for OS, and only variables with a statistical significance were listed.

TTP: time to progression; OS: Overall survival; TACE: transcatheter arterial chemoembolization; HR: hazard ratio; BCLC: Barcelona Clinic Liver Cancer; VI/EM: vascular invasion or extrahepatic metastasis.

For OS, univariate Cox regression analysis showed that subgrouping by wavelet-3-D (filter 1.0) was the only factor with a *P* value < 0.10. This also remained statistically significant in multivariate Cox regression analysis (*P* = 0.013; Table 2).

### Further validation for the consistence between endpoints

According to the results of Kaplan-Meier analysis in all the patients, Gabor-1-90 (filter 0) and wavelet-2-D (filter 1.5) performed best in identifying all the patients possibly suitable for the combination therapy. However, since wavelet-2-D (filter 1.5) did not have a statistical difference in Cox regression, Gabor-1-90 (filter 0) was superior.

Since Gabor-1-90 (filter 0) data were normally distributed between TACE-5 and TACE-6, independent *t*-tests were used for further analysis. The results showed that Gabor-1-90 was lower in the TACE-5 than in the TACE-6 group (2.9512 + 0.8190 vs. 3.5318 +0.6609, *P* = 0.012), which confirmed the consistency between Gabor-1-90 (filter 0) and wavelet-3-D (filter 1.0).

## DISCUSSION

Although studies have proven that sorafenib could suppress the elevation of VEGF after TACE and may bring a survival benefit to these patients [30, 31], this issue was still controversial [13, 14]. Furthermore, since sorafenib could increase side effects [9, 10, 13], sorafenib administration may influence liver function and disturb the schedule of TACE. Therefore, before using such combination therapy, patients should be selected to identify those who may truly experience a survival benefit. Therefore, we conducted this study and proved that some textural parameters could assist in this selection process. Specifically, Gabor-1-90 (filter 0), Gabor-1-135 (filter 1.0), Gabor-1-135 (filter 1.5), wavelet-2-D (filter 1.0), and wavelet-2-D (filter 1.5) were indicative of TTP, while wavelet-2-D (filter 1.5) was indicative of OS.

In order to identify such patients appropriately, 3 steps were designed in this study. Firstly, textural parameters conveying prognostic information were identified and patients in the TACE group were divided into lower and higher subgroups according to their thresholds. TTP and OS in the 2 TACE subgroups were compared. Secondly, the TTP and OS of the subgroups were compared to those of the TACE+S group. Multivariate Cox regression analysis was used to test whether the subgrouping represented independent prognostic factors. Thirdly, consistency was confirmed between the parameters of TTP and OS. After those steps, we proved that compared with the combination group, although some patients in the TACE group (e.g. TACE-1 and TACE-5) had similar survival, others (e.g. TACE-2 and TACE-6) indeed showed a shorter survival. Considering the comparability of baseline characteristics between the TACE+sorafenib group vs. the TACE-2 group (Gabor-1-90 > 3.6190) or TACE+sorafenib group vs. the TACE-6 group (wavelet-3-D >12.2620), as well as the consistency of the first and second outcomes, we believed that if TACE-2 or TACE-6 were treated by the combination of TACE and sorafenib, they would probably have a better survival. Thus the cohort had similar textural features with TACE-2 or TACE-6 are the suitable patients for the combination therapy.

**Figure 3:**
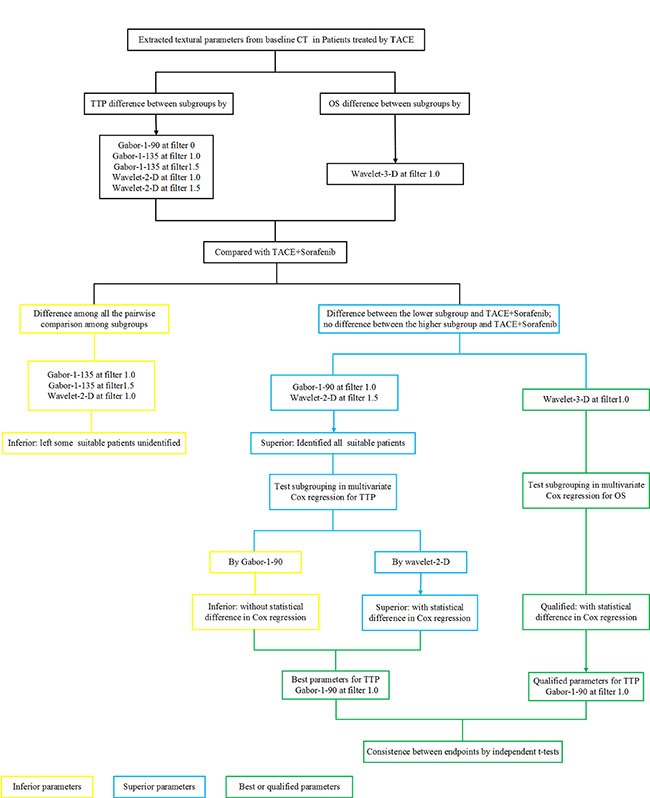
Flowchart of identifying the most suitable textural parameters

Although 5 parameters proved to be related to TTP, it is necessary to identify the best indicators in order to facilitate clinical application, and avoid contradiction by classification using different parameters. Based on the potential survival benefit afforded by sorafenib, we aimed to select the most appropriate patients. Therefore, when separated by Gabor-1-135 (filter 1.0), Gabor-1-135 (filter 1.5), or wavelet-2-D (filter 1.0), TTP still showed statistically significant differences between the lower value group (such as TACE-3) and the TACE+Sorafenib group. This indicated that some appropriate patients remained unidentified; these parameters were therefore inferior to Gabor-1-90 (filter 0) and wavelet-2-D (filter 1.5). In Cox regression analysis of the total patient group, subgrouping by wavelet-2-D (filter 1.5) did not result in statistically significant differences; therefore, Gabor-1-90 (filter 0) was the best parameter for determining benefit of TTP (Figure 3).

Since more confounders were induced after PD during treatment, OS was only used as the second endpoint. Following a similar process as for TTP, wavelet-3-D (filter 1.0) was found to be the only parameter significantly related to OS. Nevertheless, since we used 2 different parameters for TTP and OS, we performed further validation and showed that the TTP endpoint by Gabor-1-90 (filter 0) and the OS endpoint by wavelet-3-D (filter 1.0) were consistent (Figure 3).

In previous studies, radiomic on CT images has been suggested to be a potential prognostic biomarker. One seminal study identified that HCC imaging phenotypes, such as tumor margin scores, are strongly correlated with the gene expression program of the doxorubicin-response [32]. A thorough prospective radiomic analysis was performed in 1,019 patients with lung or head-and-neck cancer and showed that radiomics that included textural parameters could identify differences in the phenotypes [33]. Additionally, studies on locally advanced lung adenocarcinoma identified significant corrections between CT textural parameters and distant metastasis [34]. Moreover, in primary colorectal cancer, texture features were also associated with the 5-year OS rate [35]. These studies highlighted the potential use of texture analysis in prognosis prediction, and we consider that it could facilitate personalized treatment.

The Gabor or wavelet transforms, with their space-frequency decomposition abilities, have previously been applied in texture analysis. In the Gabor feature space, changes in object location, scale, and orientation are clearly detected [36]. The wavelet transform provides a natural adaptability to local signal properties and non-stationary signals, and thus can be used for texture characterization, segmentation, and classification [37, 38].

This study had some limitations. Firstly, as a retrospective study, possible confounding effects were present, although we attempted to control for these as far as possible; for this reason, we chose TTP rather than OS as our primary endpoint. And also theoretically, the incorporation of genomics and radiomics data may provide more comprehensive conclusion to this issue, but since biopsy was unnecessary in TACE, we could not achieve genomics data in most of the patients. Secondly, identification of patients for this combination therapy remains a highly complex matter. Therefore, the results of one study, performed at a single center, cannot be considered the final conclusion, particularly in terms of the threshold of the parameters identified. Thirdly, all regions of interest were manually drawn by the 2 radiologists; although excellent inter-observer agreement was reached, automatic segmentation would provide a more objective assessment and also save time. Fourthly, texture analysis has been performed for a limited tumor area, but not for the whole tumor; however, another study using only the largest tumor dimension have shown its promise as a predictive biomarker [21]. This could be addressed by improved software that would enable whole tumor segmentation and analysis.

In conclusion, textural parameters have proven to be promising in appropriately selecting patients for TACE and sorafenib combination treatment; in particular patients with Gabor-1-90 (filter 0) ≤ 3.8190 or wavelet-2-D (filter 1.5) ≤ 6.7515 appear to be most likely to obtain survival benefit from the combination therapy.

## MATERIALS AND METHODS

This retrospective study was approved by the Ethics Committee of Guangdong General Hospital. Informed consent was waived as all patient details were anonymized and de-identified prior to analysis.

### Patients

Between September 2007 and December 2014, patients with HCC that was initially treated by TACE, with or without sorafenib, were enrolled from Guangdong General Hospital. The diagnosis of HCC was based on non-invasive criteria, according to the recommendation of EASL-EORTC and AASLD [4, 5]. The inclusion criteria were as follows: (1) no previous treatment for HCC before initial TACE; (2) minimum follow-up of 3 years if still alive by the end of the study period (31 March 2015). Exclusion criteria were: (1) initially diagnosed based on magnetic resonance imaging (MRI) rather than computed tomography (CT); (2) irregular follow-ups; (3) accompanied by other cancers; (4) death unrelated to HCC; (5) incomplete CT data sets (1.25 mm); (6) decreased liver attenuation values on precontrast CT images, steatosis on pre-contrast CT images. Supplementary Figure S1 summarizes the process for patients’ inclusion and exclusion.

### Candidate clinical factors

Candidate clinical factors for the Cox proportion hazard model included age, sex, maximum diameter (MD), lesion number (N: 1, 2, 3, and > 4), cirrhosis (absence or presence), BCLC stage (AB, B, or C), Child−Pugh class (CP: A or B), vascular invasion or extrahepatic metastasis (VI/EM, absence or presence), alpha fetoprotein level (AFP, < 25 μg/mL, 25–400 μg/mL, or > 400 μg/mL), hepatitis infection (absence, A, B, C, D, or E).

Since biopsy is not essential for diagnosis and staging of HCC [4, 5], and as a gross specimen cannot be obtained during TACE, pathological-related data, such as TNM stage, were not available in this study. Moreover, we used the BCLC stage system instead, as recommended by the guidelines. In the BCLC staging system, considering the controversies involved in the staging of a single HCC > 5 cm, we incorporated stage AB according to our own and others’ previous studies [29, 39].

### Follow-ups

The time interval between baseline CT and initial treatment was limited to less than 2 weeks. The follow-up interval was 4–8 weeks, including routine laboratory tests, chest radiography, and abdominal CT. Additional CT was routinely added when extrahepatic metastasis was suspected. Subsequent TACE and ablation were determined by our multidisciplinary team (MDT). In general, the decisions were made in reference to treatment response, evidence from current guidelines [4, 5], and the patients’ status and intention to treatment.

### Endpoints

The primary endpoint was time to progression (TTP), which was defined as the time interval from diagnosis to disease progression (PD). PD was defined according to the mRECIST criteria [40]. The secondary endpoint was overall survival (OS), which was defined as the time interval from diagnosis to death. Considering the potential survival benefit of the combination therapy, we planned to identify the most suitable patients, either for TTP or for OS. Based on this, the textural parameters was tested for TTP and OS separately.

### CT technique

All patients had undergone conventional contrast-enhanced upper abdominal CT using the same scanner (LightSpeed VCT 64, GE Medical Systems, Waukesha, WI). The scan range was from the right diaphragmatic surface to the inferior border of the liver in the cranio-caudal direction. After administering a non-ionic contrast medium, iopamidol (370 mg of iodine/mL; Iopamiro; Bracco, Milan, Italy) at a dose of 1.5 mL/kg (maximum dose: 100 mL) with a double-tube high-pressure syringe, at a rate of 3.5 mL/s, hepatic images were acquired at a fixed time point, with the portal venues phase (PVP) at a 70-s delay. Scan parameters were as follows: 120 kV; automatic tube current modulation, 80–500 mA; collimation: 64 × 0.625; noise index, 7; pitch/table speed, 0.984/39.37 mm/rot; rotation time, 0.6 s; field of view, 300–450 mm; matrix, 512 mm. Image reconstruction was performed with a soft tissue kernel and a slice thickness of 1.25 and 5 mm.

### Texture analysis

For each pre-treatment examination, 1.25-mm DICOM format axial portal venous phase images, through the largest cross-sectional area of tumor, were selected as the region of interest and transferred to 2 personal computers for texture analysis. Two radiologists (Reader 1 and Reader 2, with 5 and 4 years of experience in abdominal CT interpretation, respectively), who were blinded to patients’ clinical and pathological information, reviewed the aforementioned images independently. The feature extraction methods of Gabor filter and wavelet analysis were used [36, 41]. The process of texture analysis comprised 3 steps, as described in our previous paper, using MATLAB 2014a software (MathWorks Inc., Natick, MA) and Image J software (National Institutes of Health, Bethesda, MD): (1) image filtration, (2) Gabor filter or wavelet transform (3) texture feature extraction [29].

### Statistical analysis

Shapiro–Wilk test was applied for normality and Levene's test for homogeneity of variance. Differences in patient demographics and characteristics between groups were tested by using 2 independent-sample *t*-tests, Mann–Whitney *U*-tests, and chi-square tests.

The clinical characteristics of TACE patients were used in adjustment. Univariate Cox regression was used as a preliminary screening for variables to reduce the size of the feature set. Factors with a *P* < 0.10 were entered into the subsequent multivariate Cox regression models (Forward: LR method). Textural parameters for each spatial filter were tested in separate Cox regression to assess the potential relationship between CT textural parameters of the primary mass and the TTP and OS respectively. Receiver operating characteristic curves (ROCs) were used to identify the thresholds. Finally, independent-sample *t*-tests or the Mann–Whitney U test, was performed to compare the identified textural parameters among the subgroups according to their normality and homogeneity.

All statistical analyses were performed with SPSS 20.0 (IBM SPSS Statistics, Armonk, NY) and R software (version 3.2.0; R Foundation for Statistical Computing, Vienna, Austria). A two-tailed *P*-value of less than 0.05 was considered statistically significant.

## SUPPLEMENTARY MATERIALS FIGURES AND TABLES






