# Supplementary material for: Texture analysis of intermediate-advanced hepatocellular carcinoma: prognosis and patients' selection of transcatheter arterial chemoembolization and sorafenib
Source: Oncotarget. 2016 Nov 29;8(23):37855–65. doi: 10.18632/oncotarget.13675 (PMC5514956; doi:10.18632/oncotarget.13675)
Supplement: Supplementary file 1 [file oncotarget-08-37855-s001.pdf]

Texture analysis of intermediate-advanced hepatocellular carcinoma: prognosis and patients' selection of transcatheter arterial chemoembolization and sorafenib

Supplementary Materials

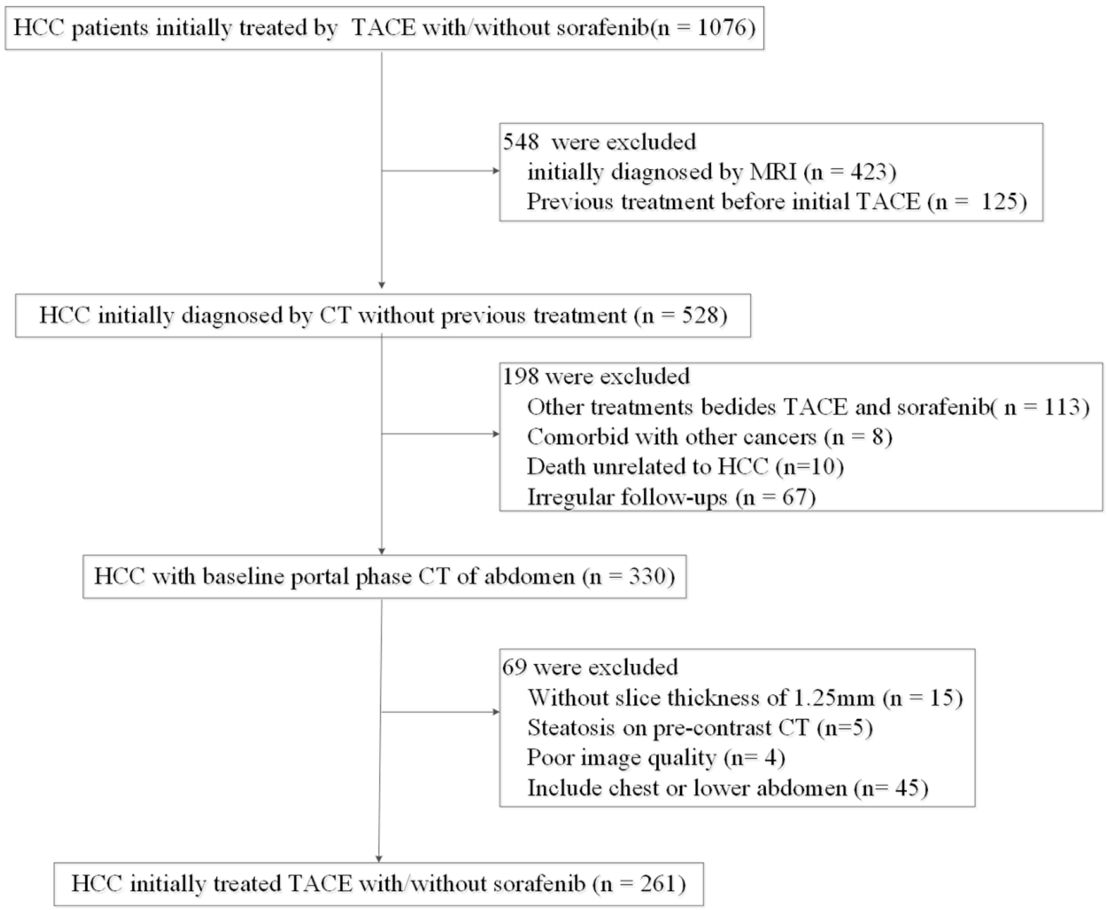

Abbreviations: HCC: hepatocellular carcinoma; CT: computer tomography; TACE: transcatheter arterial chemoembolization

Supplementary Figure S1: Flowchart of patients' inclusion and exclusion.

Supplementary Table S1: Demographic and baseline characteristics of the patients. see Supplementary_Table_S1

Supplementary Table SE1: Univariate Cox regression for textural parameters in TTP and OS in TACE patients. see Supplementary_Table_SE1

Supplementaryy Table SE2: Univariate Cox regression of clinical factors for TTP and OS in TACE patients. see Supplementary_Table_SE2

Supplementary Table SE3: Multivariate Cox regression for TTP and OS in TACE patients

	Gabor filter			Wavelet transform		
**Cox model***	**Factors**	**HR (95% CI)**	***P***	**Factors**	**HR (95% CI)**	***P***
**TTP**						
**Filter 0**	**Gabor-1-90**	1.516 (1.049–2.191)	0.027		None identified	
**Filter 1.0**	**Gabor-1-135**	2.543 (1.229–5.262)	0.012	**VI/EM**	0.548 (0.313–0.962)	0.036
				**Wavelet-2-D**	1.085 (1.022–1.153)	0.008
**Filter 1.5**	**Gabor-1-135**	5.276 (1.645–16.916)	0.005	**Wavelet-2-D**	1.119 (1.030–1.217)	0.008
**OS**						
**Filter 0**		-			-	
**Filter 1.0**		None identified		**Sex**	4.056 (0.974–16.887)	0.054
				**Wavelet-3-D**	1.121 (1.004–1.251)	0.043
**Filter 1.5**		-			-	

Abbreviations: *Twelve separate multivariate cox regression analyses were performed: 6 for TTP and 6 for OS, and only variables with a statistical significance were listed; -waived due to the absence of identified textural parameters in univariate cox regressions.

TTP: time to progression; OS: Overall survival; TACE: transcatheter arterial chemoembolization; HR: hazard ratio; BCLC: Barcelona Clinic Liver Cancer; VI/EM: vascular invasion or extrahepatic metastasis;

Supplementary Table SE4: Kaplan-Meier method and log-rank tests for subgroups by texture analysis

Groups			Median#	*P* with T+S
**TTP**				
	**T+S**		140 (120–160)	
	**TACE**		149 (110–188)	0.844
		Gabor-1-90 (filter 0) < 3.6190	199 (160–238)	0.135
		> 3.6190	89 (77–101)	< 0.001 *
		Gabor-1-135 (filter 1. 0) < 1.3995	205 (126–284)	0.009*
		> 1.3995	102 (77–127)	0.033*
		Gabor-1-135 (filter 1.5) < 0.5175	287 (189–385)	0.010*
		> 0.5175	102 (77–125)	0.016*
		Wavelet-2-D (filter 1.0) < 18.3585	244 (167–321)	0.012*
		> 18.3585	102 (88–116)	0.022*
		Wavelet-2-D (filter 1.5) < 6.7515	199 (154–244)	0.152
		> 6.7515	98 (72–124)	0.049*
**OS**				
	**T+S**		612 (549–675)	
	**TACE**		590 (524–656)	0.592
		Wavelet-3-D (filter 1.0) < 12.2620	711 (591–831)	0.072
		> 12.2620	349 (248–450)	< 0.001 *

Abbreviations: #Unit: days; *with a statistical difference; T+S: TACE+sorafenib; TTP: time to progression; OS: overall survival; TACE: transcatheter arterial chemoembolization.
