# Supplementary material for: Texture analysis of intermediate-advanced hepatocellular carcinoma: prognosis and patients' selection of transcatheter arterial chemoembolization and sorafenib
Source: Oncotarget. 2016 Nov 29;8(23):37855–65. doi: 10.18632/oncotarget.13675 (PMC5514956; doi:10.18632/oncotarget.13675)
Supplement: Supplementary file 2 [file oncotarget-08-37855-s002.docx]

| **Table 1 Demographic and baseline characteristics of the patients** | | | | |
| --- | --- | --- | --- | --- |
|  | **All (N = 261)** | **TACE (N = 197)** | **Sorafenib (N = 64)** | **P** |
| **Age** | 56 (20–83)* | 58(20–84)* | 54 (20–79)* | 0.103 |
| **Sex (N)** |  |  |  | 0.094 |
| Male | 241 | 185 | 56 |  |
| Female | 20 | 12 | 8 |  |
| **BMI (kg/m^2^)** | 24(15–32)* | 24 (15–32)* | 23 (16–33)* | 0.840 |
| **Cause of disease (N)** |  |  |  | 1.000 |
| HBV | 195 | 147 | 48 |  |
| HCV | 4 | 3 | 1 |  |
| Negative | 62 | 47 | 15 |  |
| **Child–Pugh class (N)** |  |  |  | 0.231 |
| A | 180 | 132 | 48 |  |
| B | 81 | 65 | 16 |  |
| **BCLC (N)** |  |  |  |  |
| AB |  | 62 | 13 | 0.081 |
| B |  | 75 | 26 |  |
| C |  | 60 | 25 |  |
| **Vascular invasion** |  |  |  | 0.114 |
| No | 176 | 138 | 38 |  |
| Yes | 85 | 59 | 26 |  |
| **Cirrhosis** |  |  |  | 0.984 |
| Yes | 196 | 148 | 48 |  |
| No | 65 | 49 | 16 |  |
| **MD (mm)** | 75 (42–187)* | 74 (42–187)* | 77 (48–175)* | 0.821 |
| **Lesion number (N)** |  |  |  | 0.446 |
| N = 1 | 113 | 93 | 20 |  |
| N = 2 | 67 | 33 | 34 |  |
| N = 3 | 13 | 3 | 10 |  |
| N > 4 | 68 | 68 | 0 |  |
| **Albumin (g/L)** | 35 (21–48)* | 35 (22–44)* | 34 (21–48)* | 0.568 |
| **TBIL (μmol/L)** | 20 (5–52)* | 20 (8–52)* | 22 (5–37)* | 0.812 |
| **Prothrombin time** | 14 (12–16)* | 14 (12–15)* | 14 (12–16)* | 0.418 |
| **ALT (μmol/L)** | 38 (10–566)* | 38 (15–566)* | 39 (10–236)* | 0.480 |
| **AFP (N)** |  |  |  | 0.125 |
| < 25μg/mL | 69 | 57 | 12 |  |
| 25–400 μg/mL | 93 | 71 | 22 |  |
| > 400 μg/mL | 99 | 69 | 30 |  |
| * median (range) for data without normal distribution.  Abbreviations: BCLC: Barcelona Clinic Liver Cancer; BMI: body mass index; HBV: hepatitis B virus; HCV: hepatitis C virus; MD: maximum diameter; TBIL: total bilirubin; ALT: alanine aminotransferase, AFP: alpha fetoprotein | | | | |
