# Supplementary material for: Texture analysis of intermediate-advanced hepatocellular carcinoma: prognosis and patients' selection of transcatheter arterial chemoembolization and sorafenib
Source: Oncotarget. 2016 Nov 29;8(23):37855–65. doi: 10.18632/oncotarget.13675 (PMC5514956; doi:10.18632/oncotarget.13675)
Supplement: Supplementary file 3 [file oncotarget-08-37855-s003.docx]

| **Table E1. Univariate Cox regression for textural parameters in TTP and OS in TACE patients** | | | | | | |
| --- | --- | --- | --- | --- | --- | --- |
|  | **Gabor filter** | | | **Wavelet transform** | | |
| **Cox model**^*^ | **Factors** | **HR (95% CI)** | **P** | **Factors** | **HR (95% CI)** | **P** |
| **TTP** | | | | | | |
| **Filter 0** | **Gabor-1-45** | 1.476 (0.930–2.342) | 0.098 | **Wavelet-2-D** | 1.035 (0.994–1.077) | 0.092 |
|  | **Gabor-1-90** | 1.516 (1.049–2.191) | 0.027 |  | | |
| **Filter 1.0** | **Gabor-1-90** | 1.882 (1.083–3.273) | 0.025 | **Wavelet-1-D** | 1.013 (1.018–1.196) | 0.017 |
|  | **Gabor-1-135** | 2.543 (1.229–5.262) | 0.012 | **Wavelet-1-H** | 1.104 (0.989–1.231) | 0.077 |
|  | **Gabor-2-90** | 1.651 (1.010–2.697) | 0.045 | **Wavelet-1-V** | 1.110 (1.003–1.228) | 0.044 |
|  | **Gabor-2-135** | 2.188 (1.041–4.601) | 0.039 | **Wavelet-2-D** | 1.073 (1.013–1.136) | 0.017 |
|  | **Gabor-3-135** | 8.916 (0.693–114.667) | 0.093 | **Wavelet-2-V** | 1.072 (0.989–1.161) | 0.090 |
|  |  |  |  | **Wavelet-3-D** | 1.106 (1.007–1.215) | 0.036 |
| **Filter 1.5** | **Gabor-1-45** | 3.896 (0.975–15.574) | 0.054 | **Wavelet-1-D** | 1.112 (0.999–1.239) | 0.053 |
|  | **Gabor-1-90** | 2.549 (1.133–5.733) | 0.024 | **Wavelet-1-H** | 1.135 (0.990–1.301) | 0.070 |
|  | **Gabor-1-135** | 5.276 (1.645–16.919) | 0.005 | **Wavelet-1-V** | 1.212 (1.034–1.420) | 0.017 |
|  | **Gabor-2-0** | 8.112 (0.949–69.360) | 0.056 | **Wavelet-2-D** | 1.119 (1.030–1.217) | 0.008 |
|  | **Gabor-2-45** | 4.551 (1.066–19.427) | 0.041 | **Wavelet-2-H** | 1.230 (1.034–1.463) | 0.019 |
|  | **Gabor-2-90** | 2.617 (1.150–5.958) | 0.022 | **Wavelet-2-V** | 1.173 (1.025–1.344) | 0.021 |
|  | **Gabor-2-135** | 6.260 (1.479–26.505) | 0.013 | **Wavelet-3-D** | 1.212 (1.040–1.412) | 0.014 |
|  | **Gabor-3-45** | 260.422 (0.519–130707.144) | 0.080 | **Wavelet-3-H** | 1.407 (1.054–1.880) | 0.021 |
|  | **Gabor-3-90** | 27.351 (0.793–943.623) | 0.067 | **Wavelet-3-V** | 1.578 (1.028–2.422) | 0.037 |
|  | **Gabor-3-135** | 676.375 (1.520–301073.326) | 0.036 |  |  |  |
| **OS** |  |  |  |  |  |  |
| **Filter 0** |  | None identified |  |  | None identified |  |
| **Filter 1.0** | **Gabor-1-90** | 1.622 (0.935–2.815) | 0.085 | **Wavelet-2-D** | 1.062 (0.999–1.130) | 0.059 |
|  | **Gabor-1-135** | 2.889 (1.243–6.712) | 0.014 | **Wavelet-3-D** | 1.114 (1.000–1.240) | 0.050 |
|  | **Gabor-2-135** | 2.294 (0.963–5.446) | 0.061 |  |  |  |
|  | **Gabor-3-90** | 5.153 (0.905–29.341) | 0.065 |  |  |  |
|  | **Gabor-3-135** | 14.305 (0.673–303.929) | 0.088 |  |  |  |
| **Filter 1.5** |  | None identified |  |  | None identified |  |
| ^*^Twelve separate multivariate cox regression analyses were performed, 6 for TTP and 6 for OS;  Abbreviations: TTP: time to progression; OS: Overall survival; HR: hazard ratio; | | | | | | |

| **Table E2. Univariate Cox regression of clinical factors for TTP and OS in TACE patients** | | | | |
| --- | --- | --- | --- | --- |
|  | **TTP** | | **OS** | |
| **Factors** | **HR (95% CI)** | **P** | **HR (95% CI)** | **P** |
| **Sex** | 2.453 (0.762–7.890) | 0.132 | 3.952 (0.951–16.422) | 0.059^*^ |
| **Age** | 0.995 (0.980–1.010) | 0.496 | 1.003 (0.985–1.021) | 0.721 |
| **BCLC stage**  C | Reference | 0.203 | Reference | 0.936 |
| AB | 0.562 (0.294–1.077) |  | 0.953 (0.461–1.967) |  |
| B | 0.676 (0.371–1.229) |  | 0.880 (0.433–1.788) |  |
| **VI/EM** | 0.624 (0.362–1.076) | 0.090^*^ | 0.913 (0.482–1.729) | 0.780 |
| **Maximum diameter** | 1.000 (0.995–1.006) | 0.163 | 1.005 (0.999–1.011) | 0.114 |
| **Lesion number**  N > 4 | Reference | 0.379 | Reference | 0.482 |
| N = 1 | 0.701 (0.400–1.226) |  | 0.795 (0.417–1.516) |  |
| N = 2 | 0.542 (0.260–1.129) |  | 0.507 (0.213–1.208) |  |
| N = 3 | 0.585 (0.078–4.364) |  | 0.569 (0.075–4.330) |  |
| **Cirrhosis** | 0.746 (0.453–1.228) | 0.249 | 0.832 (0.475–1.458) | 0.521 |
| **Child−Pugh Class** | 0.878 (0.520–1.481) | 0.626 | 0.706 (0.392–1.271) | 0.246 |
| **Hepatitis** HCV | Reference | 0.302 | Reference | 0.917 |
| Negative | 0.785 (0.186–3.310) |  | 0.800 (0.189–3.384) |  |
| HBV | 1.529 (0.476–4.912) |  | 0.972 (0.300–3.147) |  |
| **AFP** > 400 μg/mL | Reference | 0.255 | Reference | 0.177 |
| < 25 μg/mL | 0.620 (0.346–1.111) |  | 0.571 (0.302–1.081) |  |
| 25−400 μg/mL | 0.864 (0.448–1.664) |  | 0.590 (0.279–1.249) |  |
| **Capsule** Integral | Reference | 0.090^*^ |  | 0.372 |
| Absence | 2.183 (1.054–4.518) |  | 1.746 (0.794–3.843) |  |
| Not integral | 1.998 (0.992–4.025) |  | 1.515 (0.720–3.189) |  |
| **Shape** | 0.692 (0.407–1.174) | 0.172 | 1.204 (0.678–2.137) | 0.526 |
| **Corona** | 0.326 (0.421–1.647) | 0.585 | 0.674 (0.325–1.395) | 0.288 |
| **Mosaic** | 1.003(0.585–1.719) | 0.992 | 1.246 (0.684–2.270) | 0.471 |
| **Node in Node** | All negative |  | All negative |  |
| **Enhanced region** > 75% | Reference | 0.186 |  | 0.737 |
| Negative | 0.482 (0.116–2.007) |  | 0.639 (0.152–2.683) |  |
| 0%–25% | 0.809 (0.194–3.367) |  | 0.370 (0.050–2.709) |  |
| 25%–50% | 1.603 (0.619–4.147) |  | 0.922 (0.282–3.010) |  |
| 50%–75% | 1.971 (1.024–3.793) |  | 0.672 (0.298–1.518) |  |
| **Post-RFA** |  |  | 0.868 (0.494–1.524) | 0.622 |
| ^*^ factors in the univariate analyses with a P < 0.10 were entered into multivariate analyses.  Abbreviations: TACE: transcatheter arterial chemoembolization; HR: hazard ratio; BCLC: Barcelona Clinic Liver Cancer; VI/EM: vascular invasion or extrahepatic metastasis; HBV: hepatitis B virus; HCV: hepatitis C virus; AFP: alpha fetoprotein; PEI: percutaneous ethanol injection; RFA: Radiofrequency ablation | | | | |
